# Overweight and Obesity Determine the Risk for Gastrointestinal Cancer in a Sex-Dependent Manner: A Retrospective Cohort Study of 287,357 Outpatients in Germany

**DOI:** 10.3390/cancers14040931

**Published:** 2022-02-13

**Authors:** Sven H. Loosen, Christoph Roderburg, Markus S. Jördens, Georg Fluegen, Tom Luedde, Karel Kostev

**Affiliations:** 1 Clinic for Gastroenterology, Hepatology and Infectious Diseases, University Hospital Duesseldorf, Medical Faculty of Heinrich Heine University Duesseldorf, 40225 Duesseldorf, Germany; christoph.roderburg@med.uni-duesseldorf.de (C.R.); markus.joerdens@med.uni-duesseldorf.de (M.S.J.); 2 Department of General, Visceral, Thoracic and Pediatric Surgery (A), Medical Faculty, Hein-rich-Heine-University, University Hospital Duesseldorf, 40225 Duesseldorf, Germany; georg.fluegen@med.uni-duesseldorf.de; 3 Epidemiology, IQVIA, 60549 Frankfurt, Germany; karel.kostev@iqvia.com

**Keywords:** BMI, body mass index, fat, tumor, epidemiology, colon cancer

## Abstract

**Simple Summary:**

By analyzing a large German primary care provider database, we demonstrated that obesity represents a decisive risk factor for the development of colon, rectal, and liver cancer, partly in a sex-dependent manner. Thus, along with previous data, our study including >280,000 patients suggests that the clinical management of overweight patients should include a careful and structured risk assessment for the development of cancer in order to improve long-term outcomes in these patients.

**Abstract:**

Cancer represents the second leading cause of death worldwide, implementing a major health care and socioeconomic burden. Overweight and obesity, both of which are dramatically on the rise in both highly and less developed regions worldwide, have been established as modifiable risk factors for the development of various tumor entities including gastrointestinal (GI) cancers such as colorectal or gastric cancer. However, systematic data on an association between excessive body fat and GI cancer development from Germany are missing. Methods: A total of 287,357 adult outpatients with an available BMI value between 2010 and 2019 were identified from the Disease Analyzer database (IQVIA). The main outcome was the association between pre-obesity (BMI 25–30 kg/m^2^) and obesity (BMI ≥ 30 kg/m^2^) compared to normal weight (BMI 18.5–25 kg/m^2^) and the incident of a GI cancer diagnoses (including colon, rectum, stomach, pancreas, and liver cancer). Results: Within the observation period, the proportion of colon cancer patients increased stepwise from 0.5% and 0.64% in normal weight to 0.71% and 0.91% in obese female and male patients, respectively, which was confirmed in multivariable regression models (OR_female obesity_: 1.23; 95% CI: 1.03–1.48; OR_male obesity_: 1.43, 95% CI: 1.17–1.74). In contrast, multivariable regression models revealed that obesity was significantly associated with rectal cancer (OR: 1.36, 95% CI: 1.01–1.84) as well as liver cancer (OR: 1.79, 95% CI: 1.17–2.73) in men only. Conclusions: Our data suggest that obesity represents a decisive risk factor for the development of colon, rectal, and liver cancer, partly in a sex-dependent manner. Since overweight and obesity are modifiable risk factors, the current results may help to establish appropriate prevention and lifestyle programs to reduce both the incidence as well as the high morbidity and mortality of GI tumors in the future.

## 1. Introduction

With nearly 10 million deaths in 2020, cancer represents the second leading cause of death worldwide and represents a major health care and socioeconomic burden [1]. Several gastrointestinal (GI) cancers such as colorectal, gastric, esophagus, and liver cancer are among the 10 most common tumor entities worldwide in both men and women [1]. Moreover, most GI cancer are associated with a high morbidity and mortality rate [1].

The most sensible way to reduce the high number of cancer-associated deaths worldwide is certainly the primary prevention of cancer based on established cancer risk factors. Large-scale sequencing efforts have decisively improved the understanding of the genomic landscape of several malignancies in the last decades [2]. In addition to genetic alterations associated with increased risk for cancer development, several modifiable risk factors for cancer have been identified in recent decades [3]. Among these, overweight and obesity, which are dramatically on the rise in both highly and less developed regions worldwide [4], represent a crucial risk factor for cancer development [5,6]. Although the underlying pathophysiological mechanism have not been fully elucidated today, several studies have proven an association between pre-obesity or obesity and an increased risk for cancer development for different cancer entities including post-menopausal breast cancer, cervix, and ovarian cancer and renal cell carcinoma [7]. In line, there is a growing body of evidence suggesting an association between excessive body fat and the incidence of GI cancers such as colorectal cancer [8], gastric cancer [9], liver cancer [10], and pancreatic cancer [11]. Most of these GI cancers are among the most common tumor entities worldwide and are associated with a comparatively high mortality [1], corroborating their high global relevance. However, results, particularly in terms of sex-dependent influences, have partly remained inconclusive and data from larger patient cohorts in Germany are not available. In the present manuscript, we used a large outpatient database from Germany [12] to further dissect the association between pre-obesity and obesity and the risk of GI cancer development in male and female patients. 

## 2. Materials and Methods

### 2.1. Database

This study was based on data from the Disease Analyzer database (IQVIA), which contains drug prescriptions, diagnoses, and basic medical and demographic data obtained directly and in anonymous format from computer systems used in the practices of general practitioners and specialists in Germany [12]. The database covered about 3% of all outpatient practices in Germany. Diagnoses (according to International Classification of Diseases, 10th revision [ICD-10]), prescriptions (according to Anatomical Therapeutic Chemical [ATC] Classification system), and the quality of reported data are being monitored by IQVIA. In Germany, the sampling methods used to select physicians’ practices are appropriate for obtaining a representative database of general and specialized practices. It has previously been shown that the panel of practices included in the Disease Analyzer database is representative of general and specialized practices in Germany [12]. This database has been used in previous studies focusing on cancer [13,14].

### 2.2. Study Population

**Figure 1:**
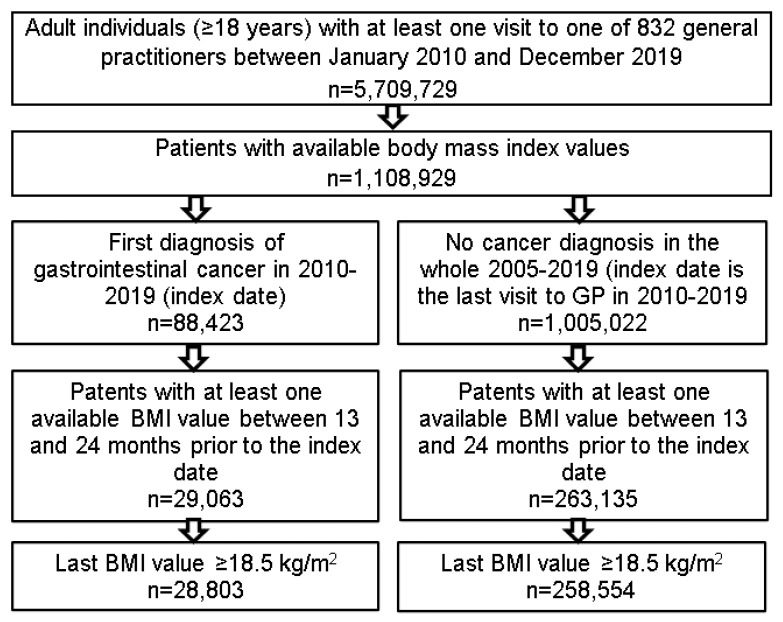
Selection of the study patients.

This retrospective cohort study included 287,357 adult individuals (≥18 years) from 832 general practices in Germany between January 2010 and December 2019. Patients were followed from January 2010 until the first diagnosis of GI cancer including colon (ICD-10: C18), rectum (ICD-10: C20), stomach (ICD-10: C16), pancreas (ICD-10: C25), and liver (ICD-10: C22) cancer (index date), or the last visit to the practice (index date), if no GI cancer was diagnosed. Patients with other cancer diagnoses were excluded from the analysis. Only patients with at least one body mass index (BMI) value within 366–730 days prior to the index date were included in the analysis (Figure 1).

### 2.3. Study Outcomes

The main outcome of this study was the association between pre-obesity (BMI ≥25 and <30 kg/m^2^) and obesity (BMI ≥30 kg/m^2^) compared to normal weight BMI (≥18.5 and <25 kg/m^2^) and the incident of a GI cancer diagnosis. For each individual, an average BMI value was calculated based on all documented BMI values within 12 to 60 months prior to the index date. BMI values within 12 months prior to the index date were not analyzed to avoid a bias caused by a BMI reduction due to an already existing but not diagnosed cancer.

### 2.4. Statistical Analyses

Differences in the sample characteristics between individuals with normal weight, overweight and obesity were tested using chi-squared tests for categorical variables and Wilcoxon tests for age. A multivariable logistic regression model was performed to study the association between the average BMI and GI cancer diagnoses. The models were conducted separately for the five cancer sites as well as for women and men to dissect potential sex-dependent associations. Models were adjusted for age, sex, and chronical diseases documented within five years prior to the index date: diabetes mellitus (ICD-10: E10-E14), thyroid gland disorders (ICD-10: E00-E07), hypertension (ICD-10: I10), ischemic heart diseases (ICD-10: I20-I25), heart failure (ICD-10: I50), lipid metabolism disorders (ICD-10: E78), chronic obstructive bronchitis or lung disease (ICD-10: J42-J44), diseases of esophagus, stomach, and duodenum (ICD-10: K20-K31), liver diseases (ICD-10: B18, K70-K77), and depression (ICD-10: F32, F33). *p*-values of <0.05 were considered statistically significant. Analyses were carried out using SAS version 9.4 (SAS Institute, Cary, NC, USA).

## 3. Results

### 3.1. Basic Characteristics of the Study Cohort

The present study included a total of 287,357 individuals of which 84,339 were classified as normal weight (BMI ≥18.5 and <25 kg/m^2^), 109,917 as overweight (pre-obese) (BMI ≥25 and <30 kg/m^2^) and 93,101 as obese (BMI ≥30 kg/m^2^). Basic characteristics of the study cohort are summarized in Table 1. There were significant differences between normal weighted, pre-obese, and obese patients in terms of most baseline characteristics. Individuals with normal weight were younger (57.9 years vs. ~64 years, *p* < 0.001), and the proportion of men was 37.9% among individuals with normal weight, but 55.9% among pre-obese patients (*p* < 0.001). The prevalence of diabetes increased from 14.6% in individual with normal weight to 51.2% in obese individuals (*p* < 0.001). The same trend was observed for hypertension, heart failure, lipid metabolism disorders, chronic obstructive bronchitis or lung disease, and liver diseases (Table 1, all *p* < 0.001).

### 3.2. Incidence Rates of Gastrointestinal Cancers in Normal Weight, Pre-Obese and Obese Outpatients in Germany

**Figure 2:**
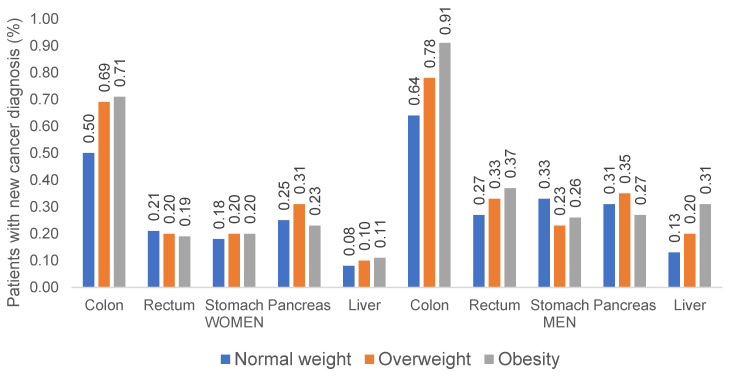
Incidence rates of gastrointestinal cancers among women and men with normal weight, overweight, and obesity.

We first compared gender-stratified incidence rates of different GI cancer sites between normal weight, pre-obese, and obese patients (Figure 2). Interestingly, we observed a stepwise increase in the proportion of colon cancer incidence from 0.5/0.64% (normal weight) to 0.71/0.91% (obese patients) in women and men, respectively (Figure 2). Multivariable regression models confirmed these results. Here, in both women (OR: 1.23; 95% CI: 1.03–1.48), and men (OR: 1.43, 95% CI: 1.17–1.74), obesity was significantly associated with an increased incidence of colon cancer (Table 2). In contrast, the incidence of rectal cancer showed an increase among pre-obese and obese male patients only, but remained comparable between normal weight, pre-obese, and obese female patients (Figure 2). In line, in the multivariable regression models, obesity was associated with rectal cancer in men only (OR: 1.36, 95% CI: 1.01–1.84, Table 2).

In terms of liver cancer, the association between an increased BMI and cancer development was most pronounced among men. Here, incidence of liver cancer increased stepwise from 0.13% in normal weight males up to 0.31% in obese men (Figure 2). Likewise, multivariable regression models showed that obesity was associated with liver cancer in men only (OR: 1.79, 95% CI: 1.17–2.73, Table 2). For gastric and pancreatic cancer, we observed a negative association between pre-obesity and stomach cancer in men (OR: 0.65, 95% CI: 0.48–0.87) and between obesity and pancreatic cancer in women (OR: 0.61, 95% CI: 0.45–0.82).

## 4. Discussion

In recent years, it has become increasingly obvious that pre-obesity and obesity represent important risk factors for the development of cancer. Both experimental and clinical analyses revealed increased rates of malignancies in patients and mice with a history of an increased body weight [3,7,8]. However, systematic data on an association between excessive body fat and GI cancer development from Germany are missing. By using the population-based Disease Analyzer database (IQVIA) [12], we demonstrated that the probability of colon, rectal, and liver cancer increases in patients with an elevated BMI, partly in a sex-dependent manner. Since pre-obesity and obesity are modifiable risk factors, the current results may help to establish appropriate prevention and lifestyle programs to reduce the high morbidity and mortality of GI tumors in the future.

Cancer represents a multifactorial disease with a complex pathophysiology. In the past, genetic causes have been extensively studied. However, it has recently become more obvious that many cancer cases are even more associated with environmental factors or a combination of both [3]. As an example, the Western dietary style, which in many cases is associated with obesity, is associated with increased cancer incidence [7]. Globally, the burden of cancer attributable to obesity, expressed as the population attributable fraction (PAF), has recently been calculated as 11.9% among men and 13.1% among women [15]. Another analysis reported that in the USA, in 2014, excess body weight accounted for 36% of gallbladder and 34% of liver cancers as well as for 32% of esophageal, 17.5% of gastric and 17% of pancreatic cancers in adults aged 30 years and older [16]. There was a positive dose-response association between excessive body weight and the risk of those cancers, which is in line with our data. In the past, various mechanisms linking obesity and pre-obesity to cancer have been proposed. Beyond the storage function of fat, adipose tissue, which is present in excess in the context of obesity, performs numerous other functions. For example, adipose tissue is an important source of hormones (so-called adipokines), growth factors, and inflammatory cytokines such as the tumor necrosis factor-α, interleukin-6 (IL-6), and others. Both TNF and IL-6 represent so called “acute phase proteins” that stimulate the liver to produce and secrete other inflammatory ligands that trigger both general and tumor-type-specific carcinogenic effects [17,18,19]. In line, various analyses have demonstrated that elevated concentrations of the C-reactive protein are associated with colorectal cancer, pancreatic cancer, endometrial malignancies, and melanoma as well as postmenopausal breast cancer and are useful to indicate treatment resistance and/or survival [20]. The fact that the adipose tissue is involved in the production and metabolism of hormones including estrogens clearly raises the question regarding sex specific effects of obesity on cancer. As an example, it was suggested that the increased risk of breast cancer among obese women might be caused by an elevated enzymatic aromatase activity of the adipose tissue, since aromatase converts androgens into estrogens [21,22,23]. Moreover, there are significant differences in adipose tissue distribution and composition among males and females that might lead to different effects of obesity even on non-sex specific cancer entities [24]. In our analyses, we found different effects of (pre-) obesity on cancer development in men and women. For example, for gastric cancer, we observed a negative association with pre-obesity in men (but not in women); similarly, in pancreatic cancer, we observed a negative association with pre-obesity in women (but not in men). Most interestingly, pre-obesity was associated with an elevated risk of colon cancer in both men and women, but with an elevated risk of rectal cancer only in men; obese women were even “protected” from the development of this entity, highlighting the complexity of the association between BMI and cancer in the different sexes. Previously, the association between hormone replacement therapy (HRT) and the risk of colorectal cancer in postmenopausal women was a topic of discussion. Previous studies reported a lower incidence of colorectal cancer in postmenopausal non-obese women under HRT [25], and an association of MSI+ colorectal cancer in obese men, but not women [26]. These and similar results such as our results here, indicate that the influence of the adipose tissue on both sex hormones and tissue homeostasis is an underappreciated risk on an epidemiological level. In this context, it is important to also note that the distribution of the gut microbiota, which has been recognized as an important risk factor for the development of GI-cancers, varies according to the patients’ sex [27]. As the gut microbiome is involved in the excretion and circulation process of sex hormones, the concept of “microgenderome” has been suggested by different authors [27] to illustrate the complex interplay of the microbiome and sex hormones that might trigger the development of cancer in female but not in men, and vice versa. Thus, our data as well as previously published data argue that patients’ sex should be taken into account when considering, for example, nutraceutics, Mediterranean diets, and/or functional foods as a possible further strategy to reduce the high morbidity and mortality of GI tumors in the future.

We acknowledge the fact that our study is limited by various aspects, most of which are due to the chosen study design and cannot be avoided [13,28,29]. Most importantly, diagnoses within our databank are recorded as ICD-10 codes, which might be associated with the misclassification of certain diagnoses. Moreover, data might be incomplete for certain patients, in particular, information regarding lab values or drug intake were not available for all patients, leading to their exclusion from this analysis (Table 1). Moreover, data on the socioeconomic status (e.g., education and income of patients) as well as lifestyle-related risk factors (e.g., smoking, alcohol consumption, and physical activity) are lacking within the Disease Analyzer database and thus cannot be taken into account here. However, the IQVIA Disease Analyzer database has been extensively published (e.g., [30,31,32]) and was proven to be representative for the outpatient sector in Germany, although only 3% of practices submit patient data to this database [12]. Additionally, we want to highlight that a selection bias must be assumed due to the fact that overweight patients presumably have more frequent physician contacts than patients who are not. Table 1 highlights that overweight patients have more concomitant disease than patients with normal body weight. Finally, subgroup analyses of individual cancer sites or subtypes (e.g., left/right sided colorectal cancer) were not feasible due to the small sample sizes, which might be associated with a presentation bias, as recently described in detail [13].

In summary, by analyzing a large German primary care provider database, we demonstrated that obesity represents a decisive risk factor for the development of colon, rectal, and liver cancer, partly in a sex-dependent manner. Thus, along with previous data, our study including >280,000 patients suggests that the clinical management of overweight patients should include a careful and structured risk assessment for the development of cancer in order to improve long-term outcomes in these patients. As an example, overweight patients might be presented in a specific interdisciplinary “metabolic board” comprising oncologists and physicians specialized in preventive medicine. In addition, these data should lead to primary prevention of tumorigenesis through obesity avoidance program as well as its therapy.

## Figures and Tables

**Table 1:** Characteristics of study patients (Disease Analyzer database, IQVIA, Germany).

Variable	All Patients	Normal Weight	Overweight	Obesity	*p* Values
N	287,357	84,339	109,917	93,101	
Age (years) (mean, SD)		57.9 (18.2)	64.6 (15.7)	64.0 (15.0)	<0.001
Age ≤50 (%)	22.8	33.4	18.5	18.1	<0.001
Age 51–60 (%)	22.1	23.1	21.6	21.8	<0.001
Age 61–70 (%)	21.3	17.0	21.6	24.7	<0.001
Age >70 (%)	33.8	26.5	38.2	35.4	<0.001
Males (%)	48.2	37.2	55.9	49.1	<0.001
Co-Diagnoses (%):					
Diabetes mellitus	32.5	14.6	30.3	51.2	<0.001
Thyroid gland disorders	21.7	31.5	30.9	32.8	<0.001
Hypertension	60.6	39.3	62.8	77.2	<0.001
Ischemic heart diseases	19.3	12.2	21.2	23.4	<0.001
Heart failure	11.6	6.7	11.5	16.3	<0.001
Lipid metabolism disorders	44.0	33.2	47.6	49.6	<0.001
Chronic obstructive bronchitis or lung disease	16.8	14.4	16.5	19.5	<0.001
Diseases of esophagus, stomach, and duodenum	42.2	40.2	43.1	43.0	<0.001
Liver diseases	15.8	9,5	16.0	21.1	<0.001
Depression	26.8	26.7	25.8	28.2	<0.001

Data are means (SD) or proportions (%).

**Table 2:** Association between overweight, obesity, and incident gastrointestinal cancers (multivariable logistic regression models).

	Odds Ratio (95% CI) ^+^				
	Colon (C18)	Rectum (C20)	Stomach (C16)	Pancreas (C25)	Liver (C22)
Women					
Overweight	1.11 (0.93–1.34)	0.82 (0.61–1.12)	0.91 (0.66–1.25)	0.87 (0.66–1.14)	0.85 (0.53–1.37)
Obesity	1.23 (1.03–1.48) *	0.83 (0.61–1.14)	0.97 (0.71–1.34)	0.61 (0.45–0.82) *	0.83 (0.51–1.35)
Men					
Overweight	1.12 (0.92–1.36)	1.11 (0.82–1.50)	0.65 (0.48–0.87) *	1.01 (0.76–1.34)	1.28 (0.84–1.95)
Obesity	1.43 (1.17–1.74) *	1.36 (1.01–1.84) *	0.79 (0.58–1.06)	0.77 (0.56–1.06)	1.79 (1.17–2.73) *

^+^ Multivariable logistic regression model adjusted for age, sex, co-diagnoses. * *p* value 0.05.

## Data Availability

The datasets used and analyzed during the current study are available from the corresponding author on reasonable request.
